# The association between hospital financial performance and the quality of care—a scoping review protocol

**DOI:** 10.1186/s13643-021-01778-3

**Published:** 2021-08-10

**Authors:** Katarzyna Dubas-Jakóbczyk, Ewa Kocot, Marzena Tambor, Wilm Quentin

**Affiliations:** 1 Health Economic and Social Security Department, Institute of Public Health, Faculty of Health Sciences, Jagiellonian University Medical College, 8 Skawińska St., 31-066, Krakow, Poland; 2 Department of Health Care Management, Technische Universität Berlin, Strasse des 17. Juni, 135 10623 Berlin, Germany; 3 European Observatory on Health Systems and Policies, WHO European Centre for Health Policy Eurostation (Office 07C020), Place Victor Horta/Victor Hortaplein, 40, /10 1060 Brussels, Belgium

**Keywords:** Hospital, Financial performance, Profit, Quality of care, Indicator

## Abstract

**Background:**

Hospitals operate under constant pressure to contain costs and improve the quality of care. The literature suggests that there is an association between health care providers’ financial performance and the quality of care. On the one hand, providers that are financially more stable might have better capacity to maintain reliable systems and resources for quality improvement. On the other hand, providing better quality of care might lead to financial gains in the form of increased revenues or achieved savings and, in consequence, a higher profitability. The general objective of this scoping review is to identify and map the available evidence on the association between hospital financial performance and the quality of care. It aims to (1) provide a broad overview of the topic and (2) indicate a more precise research question for a future systematic review.

**Methods:**

This scoping review will follow five stages: (1) defining the research question; (2) identifying relevant literature; (3) study selection; (4) data extraction; (5) collating, summarizing, and reporting the results; and (6) the consultation process and engagement of knowledge users. The following databases will be searched: MEDLINE via PubMed, (2) EMBASE, (3) Web of Science, (4) Scopus, (5) EconLit, (6) ABI/INFORM, and (7) Business Source Premier. The reference lists of relevant papers will be visually scanned with the aim of identifying further studies of interest. Also, a gray literature search will be conducted by screening the websites of diverse organizations dealing with hospital performance and/or quality of care. The review will not apply a publication date limit and will include both quantitative and qualitative empirical studies as well as theoretical papers, technical reports, books/chapters, and thesis. The reporting will utilize the PRISMA extension for a Scoping Review checklist.

**Discussion:**

This scoping review will provide an overview of the existing literature on the association between hospital financial performance and the quality of care. The review process will apply a rigorous methodological approach while broad inclusion criteria should assure comprehensive coverage of the available literature. The main limitation of the review is related to the general limitation of scoping reviews, i.e., the lack of a systematic quality and risk of bias assessment of included studies. In addition, the review will include only publications in English.

**Systematic review registration:**

Open Science Framework osf.io/z25ag

**Supplementary Information:**

The online version contains supplementary material available at 10.1186/s13643-021-01778-3.

## Background

Health systems around the world operate under constant pressure to contain costs and improve the quality of care [1–4]. Hospitals constitute a cornerstone of health service provision with their share of total current health expenditures ranging from 28.3% in Germany to 53.2% in Turkey in 2017 and being above 35% in 21 OECD countries [5]. A review by Schwierz [2] indicated that among the European Union member states a range of policy options have been implemented with the objective to contain the growth of hospital sector costs. Studies from numerous countries have indicated that hospitals might often face challenges related to long-term financial deficits (e.g., in UK [6], Italy [7], and several Central and Eastern European countries [8]) and/or risk of bankruptcy (e.g., in Germany [9] and the USA [10]). At the same time, the quality of care has been an important health policy objective at both national and international levels [3, 11]. Diverse strategies aimed at quality assurance or improvement have been implemented throughout different countries, at both system and organizational/institutional levels [3]. Several literature reviews on quality improvement strategies in hospital care can be identified [12–15], illustrating a variety of approaches in terms of the mechanisms used to improve quality as well as mixed results in terms of the effectiveness of particular approaches.

The literature suggests that there is an association between health care providers’ financial performance (FP) and the quality of care (QoC) [16]. On the one hand, providers that are more financially stable (e.g., generate profit) might provide better quality of care as they have better capacity to finance investments in new technology, pay higher wages (and/or attract more skilled staff), and maintain reliable systems and resources for quality improvement [17, 18]. On the other hand, a better quality of care might lead to financial gains in the form of increased revenues (e.g., higher reimbursement, bonuses under “pay for quality” (P4Q) programs [19]), and/or achieved savings (e.g., due to improved management or limited waste [20]). Nevertheless, the scope of available evidence on this two-way association has not been thoroughly analyzed yet.

We have identified two previous literature reviews on associations between hospital FP and QoC, yet they were focused solely on the national context of the US market [16] or conducted more than a decade ago [21]. Also, there are several published studies/reviews focusing on or including the evaluation of P4Q programs in hospital settings [19, 22–24]. Yet, in the case of those, the researchers were mainly interested in the impact of participation in the incentive program on quality of care (e.g., patient outcomes) and not on how the improvement in quality affected the overall hospital financial standing.

### Objectives

The general objective of the scoping review is to identify and map the available evidence on the association between hospital financial performance and quality of care. Both, the hospitals’ “financial performance” [25, 26] and “quality of care” [3] constitute complex and multidimensional concepts. They can be quantified by diverse individual indicators as well as some composite measures (combining multiple indicators). Following the general objectives of scoping reviews [27], we aim to provide a broad overview of the topic. We will not apply a publication date limit and will include both quantitative and qualitative empirical studies as well as theoretical papers. The results of this scoping review will help to identify and specify a more precise research topic for a future systematic review [28].

## Methods

This scoping review will be conducted based on the methodological framework outlined by Arksey and O’Malley [29] and further developed by Levac et al. [30]. This framework includes the following stages: (1) defining the research question; (2) identifying relevant literature; (3) study selection; (4) data extraction; (5) collating, summarizing, and reporting the results; and (6) the consultation process and engagement of knowledge users. The reporting will utilize the PRISMA extension for Scoping Reviews (PRISMA-ScR) checklist [31]. The search strategy will be finalized and the searches conducted in August 2021, while data screening, extraction, and synthesis will take place from September 2021 with the scoping review being finalized by late Autumn, 2021. This project has been registered through the Open Science Framework [32].

### Stage 1—Defining the research questions

In order to realize the general objective of this study, i.e., to identify and map the available evidence on the association between hospital financial performance and the quality of care, we have formulated the following specific research questions (RQ):

- RQ1—What types of studies were conducted/papers published?
- RQ2—What type of conceptual/theoretical framework was applied?
- RQ3—What type of association was being assessed?
- RQ4—How was the financial performance defined and measured?
- RQ5—How was the quality of care defined and measured?
- RQ6—What association was identified?
- RG7—What limitations were stated?

### Stage 2—Identifying relevant literature

Identification of the relevant studies will be achieved by searching the following electronic databases: (1) MEDLINE via PubMed, (2) EMBASE, (3) the Web of Science Core Collection, (4) Scopus, (5) EconLit, (6) ABI/INFORM, and (7) Business Source Premier. An initial scan demonstrated that these databases are most likely to identify publications that are related to the focus of this scoping review. The reference list of relevant papers will be visually scanned with the aim of identifying further studies of interest. Also, a gray literature search will be conducted by screening the websites of diverse international and national organizations dealing with hospital performance and/or quality of care.

The search strategy will combine terms from three topics: (1) hospital and (2) financial performance and (3) quality of care (Table 1). As both financial performance and quality of care are multidimensional concepts, the keyword formulation is challenging. The search strategy will address this issue by being iteratively developed by the research team in collaboration with an experienced librarian and experts in the field.

**Table 1:** Search terms for the databases

Topic	Search terms
Hospital	hospital* OR inpatient*
Financial performance	financial performance OR financial standing OR financial situation OR financial indicator* OR financial condition* OR financial failure OR financial distress OR financial measure* OR financial parameter* OR profit* OR operating margin* OR cash flow OR debt* OR liquidity OR asset turnover
Quality of care	quality OR staff* OR technology OR health outcome* OR patient* safety OR patient* satisfaction OR readmission* OR adverse event* OR complication*

Terms will be searched as keywords in the title and/or abstract without a publication date limit. We will use medical subject headings (MeSH and Emtree, respectively) as well as related text words. Additional file 1 presents an example of the initial search strategy conducted in MEDLINE via PubMed.

### Stage 3—Study selection

The search results will be downloaded and imported into Mendeley reference manager, which will be used for the study selection process. The selection will consist of two stages of screening: (1) a title and abstract review and (2) a full-text review. For the first level of screening, the following procedure will be applied: two researchers (authors of this protocol) will screen a random 10% sample of records and compare and discuss their results until consensus has been reached. If an agreement between them is sufficiently high (at least 80% raw agreement), the remaining records will be screened by one researcher. If the agreement is below 80%, another 10% sample will be screened by the same two researchers and the process will be repeated. The full-text articles will be assessed independently by two researchers to determine whether they meet the following inclusion criteria:

- Both FP and QoC are defined and measured
- The focus is the hospital setting
- The association between FP and QoC is assessed
- It is a peer-reviewed empirical study or theoretical paper, technical report, book/chapter, thesis
- The full text is available in English (conference abstract will not be included)

Any discrepancies between the two researchers will be addressed by consulting the third researcher who will take a final decision on paper inclusion.

### Stage 4—Data extraction

A data collection table will be developed by the research team. The data will be extracted into a standardized template—in the form of a Microsoft Office Excel spreadsheet. Table 2 presents the general overview of the data collection instrument. Each section of data extraction will be related to a specific research question with assigned codes for further analysis (where appropriate). Depending on the number and type of included publication, a separate extraction table will be developed for empirical studies (quantitative and qualitative) and theoretical papers as well as gray literature. This will be an iterative process, with the data from the first 5 studies extracted independently by two researchers (the authors of this protocol) and then compared. If necessary, the data collection instrument will be adjusted (piloting the extraction sheet). Afterwards, the data from a random sample of 10% of the studies will be extracted by the same two researchers independently and compared. Any discrepancies will be further discussed to ensure consistency. If the agreement between the two researchers is sufficiently high (at least 80% raw agreement), the data of the remaining studies will be extracted by one researcher. If the agreement is below 80%, the process will be repeated until the threshold of 80% is reached.

**Table 2:** Overview of the data extraction and coding table

Research question	Data to be extracted	Coding examples
RQ1	Authors/title	N/A
	Year of publication	• Before 1990 • 1990–1999 • 2000–2010 • 2011–2020
	Publication type	Peer-reviewed empirical study Theoretical paper Technical report Book/chapter Thesis
	Research country	N/A (list of countries)
	Research design	• Empirical study o Quantitative (longitudinal vs. cross-sectional; cohort vs. time-series; pre-post design vs. case–control; others) o Qualitative • Theoretical paper
	Characteristics of hospitals	• Number of hospitals • Public vs. private • General vs. specialist hospitals • Hospital volume • Type of wards • Sample: regional vs. national
RQ2	Conceptual/theoretical framework	N/A (framework description)
RQ3	Type of association being assessed	• Statistical method used • Control variables used • Impact of FP on QoC (FP as predictor variable) • Impact of QoC on FP (QoC as predictor variable) • Both directions
RQ4	Financial performance definition and measures	• Number of indicators; single indicators vs. composite measures • Level of FP measurement (hospital vs. patient/procedure) • Profitability (diverse measures of profit, and return on assets, equity, etc.) • Liquidity (e.g., current ratio) • Debt management (e.g., debt ratio) • Asset management (e.g., asset turnover) • Others
RQ5	Quality of care definition and measures	• Number of indicators; single indicators vs. composite measures • Disease-specific or generic quality indicators • Structure (input indicators–resources used) • Process (indicators related to care delivery) • Outcome (intermediate and final health outcomes)
RQ6	Identified association	• Result of statistical analysis (ratio, statistical significance) • Overall assessment of the association between FP and QoC o Positive o Negative o Lack of association o Mixed results
RQ7	Limitations stated	• Related to data • Related to methods • Others

### Stage 5—Collating, summarizing, and reporting the results

The collected data will be analyzed using both quantitative and qualitative (thematic analysis) methods. Table 2 presents examples of the coding themes. For both concepts, FP and QoC, we will apply classifications which already exist in the literature. In the case of the FP concept, the standard ratio analysis divides indicators into four main categories: profitability, liquidity, debt management, and asset management [33]. In the case of QoC, the classical, Donabedian framework [34] divides indicators into three categories related to the structures, processes, and outcomes of care. These classifications of indicators will be used as a starting point for data analysis. Data on the identified associations will be extracted by focusing on the particular statistical analysis results and its significance level, followed by coding the overall association between FP and QoC as positive, negative, etc. During the analysis, the research team will discuss and revise the coding template when appropriate. We plan to provide a summary overview of the results by using both tables and graphical visualizations (if appropriate). Since we aim to synthesize and describe the coverage of the evidence, we will not assess the studies’ quality [35]. For the data reporting, we will utilize the PRISMA-ScR checklist [31] (Additional file 2).

### Stage 6—The consultation process and engagement of knowledge users

The objective of this stage is to obtain additional information or further insights that might be missing in the published literature [29]. At the same time, the consultation process will help to tailor and refine preliminary results based on stakeholder needs in order to support knowledge transfer into practice [30]. More specifically, our preliminary findings will be shared with the relevant stakeholders (hospital managers and quality improvement experts, e.g., during relevant national and international conferences) in order to provide a better understanding and validity of the results. As one of our objectives is to identify the topic for a future systematic review, we hope that involving stakeholders will help to formulate the most relevant research questions.

## Discussion

This scoping review will identify and map a broad spectrum of evidence on the association between hospital financial performance and the quality of care. The review process will apply a rigorous methodological approach while wide-ranging inclusion criteria (quantitative and qualitative empirical studies as well as theoretical papers, no date limit) should assure broad coverage of the available literature. In case this protocol needs to be amended following its publication, the date, detailed description, and justification for each amendment will be reported.

There are several potential limitations to be noted. Firstly, following guidance on conducting scoping reviews [35], no quality assessment and risk of bias assessment of included studies will be conducted. Secondly, due to language limitations of the review team, only publications in English will be considered. We are also aware that there is abundance of additional factors that may influence both the hospital FP and QoC and we might identify mostly observational studies (e.g., cross-sectional or longitudinal), which may identify associations but cannot answer questions of causality. Yet, we would be still able to define the strengths of these associations and describe the control variables used.

Despite the above-mentioned limitations, we believe that the results of this scoping review will be of interest for both researchers and policy-makers at the health system level, as well as hospital managers at the micro level. By providing a systematic overview of the existing literature, we aim to build a knowledge base around the topic of associations between hospital FP and QoC. We hope to answer the question whether there is a trade-off between these two areas and define more precise research questions for future investigations (e.g., a systematic review and meta-analysis). Our findings could also indicate that the relationship depends on the context, and future research would have to place a stronger emphasis on contextual factors. The findings will be published in a peer-reviewed journal. The paper will be circulated through relevant mailing lists and social media, as well as diverse research platforms. The findings will also be disseminated through conference presentations as well as summaries for key stakeholders (e.g., via the knowledge transfer platform of the leading author university).

## Supplementary Information

**Additional file 1..** Example of the initial search strategy conducted in Medline via PubMed

**Additional file 2..** PRISMA-ScR checklist

## Data Availability

All data generated or analyzed during the study are included in the manuscript.

## Abbreviations

FP: Financial performance
QoC: Quality of care
P4Q: Pay for quality
RQ: Research question

## References

[1] Hussey PS, Wertheimer S, Mehrotra A (2013). The association between health care quality and cost: a systematic review. Ann Intern Med..

[2] Schwierz C. Cost-containment policies in hospital expenditure in the European Union. European Commission. European Economy Discussion Paper 037. 016. 10.2765/253237.

[3] Busse R, Panteli QW. An introduction to healthcare quality: defining and explaining its role in health systems. In: Busse R, Klazinga N, Panteli D, Quentin W, editors. Improving heathcare quality in Europe. The European Observatory on Health Systems and Policies and OECD 2019. https://apps.who.int/iris/bitstream/handle/10665/327356/9789289051750-eng.pdf. Accessed 17 Nov 2020. 31721544

[4] Stadhouders N, Kruse F, Tanke M (2019). Effective healthcare cost-containment policies: a systematic review. Health Policy.

[5] OECD 2020 Health Statistics. https://www.oecd.org/els/health-systems/health-data.htm.

[6] The Royal College of Physicians. Underfunded, underdoctored, overstretched: the NHS in 2016. 2016. https://www.rcplondon.ac.uk/guidelines-policy/underfunded-underdoctored-overstretched-nhs-2016.

[7] Mauro M, Maresso A, Guglielmo A (2017). Health decentralization at a dead-end: towards new recovery plans for Italian hospitals. Health Policy.

[8] Dubas-Jakóbczyk K, Albreht T, Behmane D (2020). Hospital reforms in 11 Central and Eastern European countries between 2008 and 2019: a comparative analysis. Health Policy.

[9] Tuffs A (2006). One in three German hospitals faces bankruptcy. BMJ.

[10] Landry AY, Landry RJ 3rd. Factors associated with hospital bankruptcies: a political and economic framework. J Healthc Manag. 2009;54(4):252–71; discussion 271-2. 19681358

[11] World Health Organization. Handbook for national quality policy and strategy – a practical approach for developing policy and strategy to improve quality of care. In: . Geneva: World Health Organization. https://www.who.int/servicedeliverysafety/areas/qhc/nqps_handbook/en/. Accessed 17 Nov 2020.

[12] Ayanian JZ, Weissman JS (2002). Teaching hospitals and quality of care: a review of the literature. Milbank Q.

[13] Conry MC, Humphries N, Morgan K (2012). A 10 year (2000–2010) systematic review of interventions to improve quality of care in hospitals. BMC Health Serv Res.

[14] Aljuaid M, Mannan F, Chaudhry Z (2016). Quality of care in university hospitals in Saudi Arabia: a systematic review. BMJ Open.

[15] Avia I, Sri Hariyati T. Impact of hospital accreditation on quality of care: a literature review. Enfermería Clínica, Volume 29, Supplement 2, 2019, Pages 315-320, 10.1016/j.enfcli.2019.06.003

[16] Barnes M, Oner N, Ray MN, Zengul FD. Exploring the association between quality and financial performance in U.S. hospitals: a systematic review. J Health Care Finance. 2017;44(2) http://www.healthfinancejournal.com/index.php/johcf/article/view/144. Accessed 17 Nov 2020.

[17] Dong GN (2015). Performing well in financial management and quality of care: evidence from hospital process measures for treatment of cardiovascular disease. BMC Health Serv Res.

[18] Akinleye DD, McNutt LA, Lazariu V, McLaughlin C (2019). Correlation between hospital finances and quality and safety of patient care. PLoS One.

[19] Eckhardt H, Smith P, Quentin W. Pay for Quality: using financial incentives to improve quality of care. In: Busse R, Klazinga N, Panteli D, Quentin W, editors. Improving heathcare quality in Europe. The European Observatory on Health Systems and Policies and OECD 2019. https://apps.who.int/iris/bitstream/handle/10665/327356/9789289051750-eng.pdf. Accessed 17 Nov 2020.

[20] OECD (2017). Tackling wasteful spending on health.

[21] Beauvais B, Wells R (2006). Does money really matter? A review of the literature on the relationships between healthcare organization finances and quality. Hosp Top.

[22] Cashin C, Chi Y-L, Smith P et. al. Paying for performance in health care. The European Observatory on Health Systems and Policies and OECD. Open University Press 2014 http://www.euro.who.int/__data/assets/pdf_file/0020/271073/Paying-for-Performance-in-Health-Care.pdf. Accessed 17 Nov 2020.

[23] Milstein R, Schreyoegg J. Pay for performance in the inpatient sector: a review of 34 P4P programs in 14 OECD countries. Health Policy. 2016;120(10). 10.1016/j.healthpol.2016.08.009. 10.1016/j.healthpol.2016.08.009 27745916

[24] Mendelson A, Kondo K, Damberg C (2017). The effects of pay-for-performance programs on health, health care use, and processes of care: a systematic review. Ann Intern Med.

[25] Burkhardt JH, Wheeler JR (2013). Examining financial performance indicators for acute care hospitals. J Health Care Finance..

[26] Oner N, Zengul FD, Ozaydin B (2016). Organizational and environmental factors associated with hospital financial performance: a systematic review. J Health Care Finance.

[27] Tricco AC, Lillie E, Zarin W (2016). A scoping review on the conduct and reporting of scoping reviews. BMC Med Res Methodol.

[28] Munn Z, Peters MDJ, Stern C (2018). Systematic review or scoping review? Guidance for authors when choosing between a systematic or scoping review approach. BMC Med Res Methodol.

[29] Arksey H, O'Malley L (2005). Scoping studies: towards a methodological framework. Int J Soc Res Methodol.

[30] Levac D, Colquhoun H, O’Brien K (2010). Scoping studies: advancing the methodology. Implementation Sci.

[31] Tricco AC, Lillie E, Zarin W, et al. PRISMA extension for scoping reviews (PRISMA-ScR): checklist and explanation. Ann Intern Med. 2018. 10.7326/M18-0850. 10.7326/M18-0850 30178033

[32] Open Science Framework project registration: https://osf.io/9hn7u. Accessed 17 Nov 2020.

[33] Gapenski LC, Pink GH (2015). Understanding healthcare financial management.

[34] Donabedian A (1988). The quality of care. How can it be assessed?. JAMA.

[35] Peters MDJ, Godfrey CM, Khalil H (2015). Guidance for conducting systematic scoping reviews. Int J Evid Based Healthc..

