# Supplementary material for: The association between hospital financial performance and the quality of care—a scoping review protocol
Source: Syst Rev. 2021 Aug 10;10:221. doi: 10.1186/s13643-021-01778-3 (PMC8359611; doi:10.1186/s13643-021-01778-3)
Supplement: Supplementary file 1 — Additional file 1.. Example of the initial search strategy conducted in Medline via PubMed [file 13643_2021_1778_MOESM1_ESM.docx]

**Supplementary file 1 - Search strategy in Medline via PubMed**

**Date: 17.06.2021**

| **Search / Query** | **Results** |
| --- | --- |
| #32 |  |

| Search: **((((hospital*[Title/Abstract]) OR (inpatient*[Title/Abstract])) OR (administration, hospital[MeSH Terms])) AND (((((((((((((((financial performance[Title/Abstract]) OR (financial standing[Title/Abstract])) OR (financial indicator*[Title/Abstract])) OR (financial condition*[Title/Abstract])) OR (financial failure[Title/Abstract])) OR (financial distress[Title/Abstract])) OR (financial measure*[Title/Abstract])) OR (financial parameter*[Title/Abstract])) OR (profit*[Title/Abstract])) OR (operating margin*[Title/Abstract])) OR (cash flow[Title/Abstract])) OR (debt*[Title/Abstract])) OR (liquidity[Title/Abstract])) OR (asset turnover[Title/Abstract])) OR (financial situation[Title/Abstract]))) AND ((((((((((quality[Title/Abstract]) OR (assessment, healthcare quality[MeSH Terms])) OR (staff*[Title/Abstract])) OR (technology[Title/Abstract])) OR (health outcome*[Title/Abstract])) OR (patient* safety[Title/Abstract])) OR (patient* satisfaction[Title/Abstract])) OR (readmission*[Title/Abstract])) OR (adverse event*[Title/Abstract])) OR (complication*[Title/Abstract]))** | [2,741](https://pubmed-1ncbi-1nlm-1nih-1gov-1nb5yeisb00c5.hanproxy.cm-uj.krakow.pl/?term=%28%28%28%28hospital%2A%5BTitle%2FAbstract%5D%29+OR+%28inpatient%2A%5BTitle%2FAbstract%5D%29%29+OR+%28administration%2C+hospital%5BMeSH+Terms%5D%29%29+AND+%28%28%28%28%28%28%28%28%28%28%28%28%28%28%28financial+performance%5BTitle%2FAbstract%5D%29+OR+%28financial+standing%5BTitle%2FAbstract%5D%29%29+OR+%28financial+indicator%2A%5BTitle%2FAbstract%5D%29%29+OR+%28financial+condition%2A%5BTitle%2FAbstract%5D%29%29+OR+%28financial+failure%5BTitle%2FAbstract%5D%29%29+OR+%28financial+distress%5BTitle%2FAbstract%5D%29%29+OR+%28financial+measure%2A%5BTitle%2FAbstract%5D%29%29+OR+%28financial+parameter%2A%5BTitle%2FAbstract%5D%29%29+OR+%28profit%2A%5BTitle%2FAbstract%5D%29%29+OR+%28operating+margin%2A%5BTitle%2FAbstract%5D%29%29+OR+%28cash+flow%5BTitle%2FAbstract%5D%29%29+OR+%28debt%2A%5BTitle%2FAbstract%5D%29%29+OR+%28liquidity%5BTitle%2FAbstract%5D%29%29+OR+%28asset+turnover%5BTitle%2FAbstract%5D%29%29+OR+%28financial+situation%5BTitle%2FAbstract%5D%29%29%29+AND+%28%28%28%28%28%28%28%28%28%28quality%5BTitle%2FAbstract%5D%29+OR+%28assessment%2C+healthcare+quality%5BMeSH+Terms%5D%29%29+OR+%28staff%2A%5BTitle%2FAbstract%5D%29%29+OR+%28technology%5BTitle%2FAbstract%5D%29%29+OR+%28health+outcome%2A%5BTitle%2FAbstract%5D%29%29+OR+%28patient%2A+safety%5BTitle%2FAbstract%5D%29%29+OR+%28patient%2A+satisfaction%5BTitle%2FAbstract%5D%29%29+OR+%28readmission%2A%5BTitle%2FAbstract%5D%29%29+OR+%28adverse+event%2A%5BTitle%2FAbstract%5D%29%29+OR+%28complication%2A%5BTitle%2FAbstract%5D%29%29&sort=) |
| --- | --- |
| #31 |  |

| Search: **(((((((((quality[Title/Abstract]) OR (assessment, healthcare quality[MeSH Terms])) OR (staff*[Title/Abstract])) OR (technology[Title/Abstract])) OR (health outcome*[Title/Abstract])) OR (patient* safety[Title/Abstract])) OR (patient* satisfaction[Title/Abstract])) OR (readmission*[Title/Abstract])) OR (adverse event*[Title/Abstract])) OR (complication*[Title/Abstract])** | [3,201,981](https://pubmed-1ncbi-1nlm-1nih-1gov-1nb5yeisb00c5.hanproxy.cm-uj.krakow.pl/?term=%28%28%28%28%28%28%28%28%28quality%5BTitle%2FAbstract%5D%29+OR+%28assessment%2C+healthcare+quality%5BMeSH+Terms%5D%29%29+OR+%28staff%2A%5BTitle%2FAbstract%5D%29%29+OR+%28technology%5BTitle%2FAbstract%5D%29%29+OR+%28health+outcome%2A%5BTitle%2FAbstract%5D%29%29+OR+%28patient%2A+safety%5BTitle%2FAbstract%5D%29%29+OR+%28patient%2A+satisfaction%5BTitle%2FAbstract%5D%29%29+OR+%28readmission%2A%5BTitle%2FAbstract%5D%29%29+OR+%28adverse+event%2A%5BTitle%2FAbstract%5D%29%29+OR+%28complication%2A%5BTitle%2FAbstract%5D%29&sort=) |
| --- | --- |
| #30 |  |

| Search: **complication*[Title/Abstract]** | [1,045,305](https://pubmed-1ncbi-1nlm-1nih-1gov-1nb5yeisb00c5.hanproxy.cm-uj.krakow.pl/?term=complication%2A%5BTitle%2FAbstract%5D&sort=) |
| --- | --- |
| #29 |  |

| Search: **adverse event*[Title/Abstract]** | [179,099](https://pubmed-1ncbi-1nlm-1nih-1gov-1nb5yeisb00c5.hanproxy.cm-uj.krakow.pl/?term=adverse+event%2A%5BTitle%2FAbstract%5D&sort=) |
| --- | --- |
| #28 |  |

| Search: **readmission*[Title/Abstract]** | [33,962](https://pubmed-1ncbi-1nlm-1nih-1gov-1nb5yeisb00c5.hanproxy.cm-uj.krakow.pl/?term=readmission%2A%5BTitle%2FAbstract%5D&sort=) |
| --- | --- |
| #27 |  |

| Search: **patient* satisfaction[Title/Abstract]** | [100,098](https://pubmed-1ncbi-1nlm-1nih-1gov-1nb5yeisb00c5.hanproxy.cm-uj.krakow.pl/?term=patient%2A+satisfaction%5BTitle%2FAbstract%5D&sort=) |
| --- | --- |
| #26 |  |

| Search: **patient* safety[Title/Abstract]** | [328,300](https://pubmed-1ncbi-1nlm-1nih-1gov-1nb5yeisb00c5.hanproxy.cm-uj.krakow.pl/?term=patient%2A+safety%5BTitle%2FAbstract%5D&sort=) |
| --- | --- |
| #25 |  |

| Search: **health outcome*[Title/Abstract]** | [60,000](https://pubmed-1ncbi-1nlm-1nih-1gov-1nb5yeisb00c5.hanproxy.cm-uj.krakow.pl/?term=health+outcome%2A%5BTitle%2FAbstract%5D&sort=) |
| --- | --- |
| #24 |  |

| Search: **technology[Title/Abstract]** | [357,323](https://pubmed-1ncbi-1nlm-1nih-1gov-1nb5yeisb00c5.hanproxy.cm-uj.krakow.pl/?term=technology%5BTitle%2FAbstract%5D&sort=) |
| --- | --- |
| #23 |  |

| Search: **staff*[Title/Abstract]** | [180,608](https://pubmed-1ncbi-1nlm-1nih-1gov-1nb5yeisb00c5.hanproxy.cm-uj.krakow.pl/?term=staff%2A%5BTitle%2FAbstract%5D&sort=) |
| --- | --- |
| #22 |  |

| Search: **assessment, healthcare quality[MeSH Terms]** | [339,242](https://pubmed-1ncbi-1nlm-1nih-1gov-1nb5yeisb00c5.hanproxy.cm-uj.krakow.pl/?term=assessment%2C+healthcare+quality%5BMeSH+Terms%5D&sort=) |
| --- | --- |
| #21 |  |

| Search: **quality[Title/Abstract]** | [1,126,485](https://pubmed-1ncbi-1nlm-1nih-1gov-1nb5yeisb00c5.hanproxy.cm-uj.krakow.pl/?term=quality%5BTitle%2FAbstract%5D&sort=) |
| --- | --- |
| #20 |  |

| Search: **((((((((((((((financial performance[Title/Abstract]) OR (financial standing[Title/Abstract])) OR (financial indicator*[Title/Abstract])) OR (financial condition*[Title/Abstract])) OR (financial failure[Title/Abstract])) OR (financial distress[Title/Abstract])) OR (financial measure*[Title/Abstract])) OR (financial parameter*[Title/Abstract])) OR (profit*[Title/Abstract])) OR (operating margin*[Title/Abstract])) OR (cash flow[Title/Abstract])) OR (debt*[Title/Abstract])) OR (liquidity[Title/Abstract])) OR (asset turnover[Title/Abstract])) OR (financial situation[Title/Abstract])** | [36,232](https://pubmed-1ncbi-1nlm-1nih-1gov-1nb5yeisb00c5.hanproxy.cm-uj.krakow.pl/?term=%28%28%28%28%28%28%28%28%28%28%28%28%28%28financial+performance%5BTitle%2FAbstract%5D%29+OR+%28financial+standing%5BTitle%2FAbstract%5D%29%29+OR+%28financial+indicator%2A%5BTitle%2FAbstract%5D%29%29+OR+%28financial+condition%2A%5BTitle%2FAbstract%5D%29%29+OR+%28financial+failure%5BTitle%2FAbstract%5D%29%29+OR+%28financial+distress%5BTitle%2FAbstract%5D%29%29+OR+%28financial+measure%2A%5BTitle%2FAbstract%5D%29%29+OR+%28financial+parameter%2A%5BTitle%2FAbstract%5D%29%29+OR+%28profit%2A%5BTitle%2FAbstract%5D%29%29+OR+%28operating+margin%2A%5BTitle%2FAbstract%5D%29%29+OR+%28cash+flow%5BTitle%2FAbstract%5D%29%29+OR+%28debt%2A%5BTitle%2FAbstract%5D%29%29+OR+%28liquidity%5BTitle%2FAbstract%5D%29%29+OR+%28asset+turnover%5BTitle%2FAbstract%5D%29%29+OR+%28financial+situation%5BTitle%2FAbstract%5D%29&sort=) |
| --- | --- |
| #19 |  |

| Search: **asset turnover[Title/Abstract]** | [11](https://pubmed-1ncbi-1nlm-1nih-1gov-1nb5yeisb00c5.hanproxy.cm-uj.krakow.pl/?term=asset+turnover%5BTitle%2FAbstract%5D&sort=) |
| --- | --- |
| #18 |  |

| Search: **liquidity[Title/Abstract]** | [357](https://pubmed-1ncbi-1nlm-1nih-1gov-1nb5yeisb00c5.hanproxy.cm-uj.krakow.pl/?term=liquidity%5BTitle%2FAbstract%5D&sort=) |
| --- | --- |
| #17 |  |

| Search: **debt*[Title/Abstract]** | [5,359](https://pubmed-1ncbi-1nlm-1nih-1gov-1nb5yeisb00c5.hanproxy.cm-uj.krakow.pl/?term=debt%2A%5BTitle%2FAbstract%5D&sort=) |
| --- | --- |
| #16 |  |

| Search: **cash flow[Title/Abstract]** | [597](https://pubmed-1ncbi-1nlm-1nih-1gov-1nb5yeisb00c5.hanproxy.cm-uj.krakow.pl/?term=cash+flow%5BTitle%2FAbstract%5D&sort=) |
| --- | --- |
| #15 |  |

| Search: **operating margin*[Title/Abstract]** | [254](https://pubmed-1ncbi-1nlm-1nih-1gov-1nb5yeisb00c5.hanproxy.cm-uj.krakow.pl/?term=operating+margin%2A%5BTitle%2FAbstract%5D&sort=) |
| --- | --- |
| #14 |  |

| Search: **profit*[Title/Abstract]** | [27,813](https://pubmed-1ncbi-1nlm-1nih-1gov-1nb5yeisb00c5.hanproxy.cm-uj.krakow.pl/?term=profit%2A%5BTitle%2FAbstract%5D&sort=) |
| --- | --- |
| #13 |  |

| Search: **financial parameter*[Title/Abstract]** | [39](https://pubmed-1ncbi-1nlm-1nih-1gov-1nb5yeisb00c5.hanproxy.cm-uj.krakow.pl/?term=financial+parameter%2A%5BTitle%2FAbstract%5D&sort=) |
| --- | --- |
| #12 |  |

| Search: **financial measure*[Title/Abstract]** | [92](https://pubmed-1ncbi-1nlm-1nih-1gov-1nb5yeisb00c5.hanproxy.cm-uj.krakow.pl/?term=financial+measure%2A%5BTitle%2FAbstract%5D&sort=) |
| --- | --- |
| #11 |  |

| Search: **financial distress[Title/Abstract]** | [337](https://pubmed-1ncbi-1nlm-1nih-1gov-1nb5yeisb00c5.hanproxy.cm-uj.krakow.pl/?term=financial+distress%5BTitle%2FAbstract%5D&sort=) |
| --- | --- |
| #10 |  |

| Search: **financial failure[Title/Abstract]** | [17](https://pubmed-1ncbi-1nlm-1nih-1gov-1nb5yeisb00c5.hanproxy.cm-uj.krakow.pl/?term=financial+failure%5BTitle%2FAbstract%5D&sort=) |
| --- | --- |
| #9 |  |

| Search: **financial condition*[Title/Abstract]** | [343](https://pubmed-1ncbi-1nlm-1nih-1gov-1nb5yeisb00c5.hanproxy.cm-uj.krakow.pl/?term=financial+condition%2A%5BTitle%2FAbstract%5D&sort=) |
| --- | --- |
| #8 |  |

| Search: **financial indicator*[Title/Abstract]** | [129](https://pubmed-1ncbi-1nlm-1nih-1gov-1nb5yeisb00c5.hanproxy.cm-uj.krakow.pl/?term=financial+indicator%2A%5BTitle%2FAbstract%5D&sort=) |
| --- | --- |
| #7 |  |

| Search: **financial situation[Title/Abstract]** | [877](https://pubmed-1ncbi-1nlm-1nih-1gov-1nb5yeisb00c5.hanproxy.cm-uj.krakow.pl/?term=financial+situation%5BTitle%2FAbstract%5D&sort=) |
| --- | --- |
| #6 |  |

| Search: **financial standing[Title/Abstract]** | [44](https://pubmed-1ncbi-1nlm-1nih-1gov-1nb5yeisb00c5.hanproxy.cm-uj.krakow.pl/?term=financial+standing%5BTitle%2FAbstract%5D&sort=) |
| --- | --- |
| #5 |  |

| Search: **financial performance[Title/Abstract]** | [978](https://pubmed-1ncbi-1nlm-1nih-1gov-1nb5yeisb00c5.hanproxy.cm-uj.krakow.pl/?term=financial+performance%5BTitle%2FAbstract%5D&sort=) |
| --- | --- |
| #4 |  |

| Search: **((hospital*[Title/Abstract]) OR (inpatient*[Title/Abstract])) OR (administration, hospital[MeSH Terms])** | [1,609,484](https://pubmed-1ncbi-1nlm-1nih-1gov-1nb5yeisb00c5.hanproxy.cm-uj.krakow.pl/?term=%28%28hospital%2A%5BTitle%2FAbstract%5D%29+OR+%28inpatient%2A%5BTitle%2FAbstract%5D%29%29+OR+%28administration%2C+hospital%5BMeSH+Terms%5D%29&sort=) |
| --- | --- |
| #3 |  |

| Search: **administration, hospital[MeSH Terms]** | [267,890](https://pubmed-1ncbi-1nlm-1nih-1gov-1nb5yeisb00c5.hanproxy.cm-uj.krakow.pl/?term=administration%2C+hospital%5BMeSH+Terms%5D&sort=) |
| --- | --- |
| #2 |  |

| Search: **inpatient*[Title/Abstract]** | [130,098](https://pubmed-1ncbi-1nlm-1nih-1gov-1nb5yeisb00c5.hanproxy.cm-uj.krakow.pl/?term=inpatient%2A%5BTitle%2FAbstract%5D&sort=) |
| --- | --- |
| #1 |  |

| Search: **hospital*[Title/Abstract]** | [1,396,272](https://pubmed-1ncbi-1nlm-1nih-1gov-1nb5yeisb00c5.hanproxy.cm-uj.krakow.pl/?term=hospital%2A%5BTitle%2FAbstract%5D&sort=) |
| --- | --- |

Początek formularza

Dół formularza
